# What knowledge is needed? Teaching undergraduate medical students to “go upstream” and advocate on social determinants of health

**DOI:** 10.36834/cmej.58424

**Published:** 2020-03-16

**Authors:** Kate Hayman, Mei Wen, Farooq Khan, Tracey Mann, Andrew D. Pinto, Stella L. Ng

**Affiliations:** 1 Division of Emergency Medicine, University Health Network, Ontario, Canada; 2 Faculty of Medicine, University of Toronto, Ontario, Canada; 3 The Upstream Lab, Centre for Urban Health Solutions, Li Ka Shing Knowledge Institute, St. Michael’s Hospital, Ontario, Canada; 4 Department of Family and Community Medicine, St. Michael’s Hospital, Ontario, Canada; 5 Department of Family and Community Medicine, University of Toronto, Ontario, Canada; 6 Dalla Lanna School of Public Health, University of Toronto, Ontario, Canada; 7 Centre for Faculty Development, St. Michael’s Hospital, Ontario, Canada; 8 The Wilson Centre, Faculty of Medicine, University of Toronto, Ontario, Canada

## Abstract

**Background:**

We rarely teach medical students the skills required to engage in policy change to address the structural factors that underpin the social determinants of health, which are driven by the unequal distribution of power and resources in society. Acquiring the knowledge and skills to influence policy can empower students to *act on* healthcare inequities rather than simply be aware of them.

**Methods:**

Using Metzl and Hansen’s structural competency framework, we designed and piloted an intervention for medical students. Participants attended a workshop, presented to a hypothetical political stakeholder, and wrote anopinion editorial piece. Students participated in a focus group that was audio-recorded and transcribed. We coded and analyzed presentations, editorials, and transcripts to develop a thematic analysis.

**Results:**

Nine students participated in the workshop. They chose structural interventions and presented potential solutions to structural barriers in written and oral outputs. Students identified a lack of knowledge about health and political systems as a potential barrier to future advocacy work.

**Conclusion:**

Medical trainees require training in specific advocacy skills such as oral and written communication, however this alone may be insufficient. As future advocates, trainees must also acquire a specific skill set and associated knowledge about health systems and policy to navigate the systems in which they will practice.

## Abstract (fr)

**Contexte:**

Nous enseignons rarement aux étudiants en médecine les habiletésnécessaires pour participer aux changements de politique afin d’aborder les facteurs structurels sous-jacents aux déterminants sociaux de la santé, et la distribution inégale du pouvoir et des ressources dans la société. L’acquisition de connaissances et d’habiletés aptes à influencer les politiques peut encourager les étudiants à *s’engager*pour contrer les inégalités en matière de soins de santé plutôt que d’en être simplement conscients.

**Méthodes:**

À l’aide du cadre de compétence structurelle de Metzl et Hansen, nous avons conçu et pilotéune intervention pour les étudiants en médecine. Les participants ont assisté à un atelier présenté à un décideur politique simulé pour l'occasion, à la suite duquel ilsontrédigé un article d’opinion. Les étudiants ont participé à un groupe de discussion qui a été enregistré et transcrite. Nous avons codifié et analysé les présentations, les articles d’opinion, et les transcriptions pour développer une analyse thématique.

**Résultats:**

Neuf étudiants ont participé à l’atelier. Ils ont choisi des interventions structurelles et présenté des solutions potentielles aux obstacles structurels dans leurs épreuves écrites ou orales. Les étudiants ont indiqué que le manque de connaissances sur le système de santé et le système politique représente un obstacle potentiel au futur travail de défenseur.

**Conclusion:**

Les stagiaires en médecine ont besoin de formation spécifique sur les habiletés de communication orale et écrite pour se porter à la défense des patients; cependant, cette formation à elle seule peut s’avérer insuffisante. En tant que futurs promoteur de la santé, les stagiaires doivent également acquérir des habiletésspécifiques et des connaissances associées aux systèmes et aux politiques de santé pour naviguer à travers les systèmes dans lequel ils exerceront leur pratique.

## Background

Upon completion of training, Canadians expect our physicians to respond to community needs and advocate for change both within and beyond the immediate clinical environment.^1^ Effective advocacy requires knowledge in addition to the traditional medical repertoire including skills that must be taught and practiced.^2^ However, we rarely teach students the knowledge and skills necessary to engage in policy change that would improve the social determinants of health (SDOH) of patients.^3^ When we teach the SDOH as content to be learned, rather than inequitable conditions to remediate, educators risk perpetuating the inequities they may seek to change.^3^ Acquiring the knowledge and skills to advocate and influence policy might prepare students to *act on* healthcare inequities in an informed manner, rather than simply *to know* them.^4^^,^^5^

Prior studies have described curricular interventions designed to teach advocacy skills such as editorial writing^6^ and writing letters to the editor.^7^ However, researchers did not examine the content or the quality of the written pieces. Also, the topics that students chose (e.g. pool safety, choking hazards, vaccine refusal) suggested that their understanding of advocacy was focused on (individual) behavior change rather than addressing the structural determinants or resolving inequities.^7^^,^^8^ Other efforts to teach advocacy, such as Political Action Days, teach students how to influence elected officials around concerns of the profession (e.g. medical student debt). These sometimes do not focus on addressing health inequities.^9^

Based on the current gaps in advocacy education—an identified basis of knowledge and skill, and a lack of attention to systemic and structural factors—we chose to develop an educational initiative based on Metzl and Hansen’s Structural Competency paradigm. This initiative includes five intersecting knowledge and skill-sets, including recognizing the structures that shape clinical interactions, developing an extra-clinical language of structure, rearticulating “cultural” formulations in structural terms, observing and imagining structural interventions, and developing structural humility.^10^ This framework has been utilized by educators in pre-health curriculum,^11^ pre-clinical education,^12^ and as a checklist by clinical trainees.^13^ Within this framework, we developed and piloted a brief curricular intervention to allow medical students to practice advocacy.

## Methods

Our study protocol received approval from the University Health Network Research Ethics Board (17-5068). Our goal was to recruit a convenience sample of second year (pre-clinical) medical students at the University of Toronto (Toronto, Canada) to participate. We obtained written consent from each participant prior to participation in the study. Using Metzl and Hansen’s structural competency framework, we designed and piloted a three-hour workshop designed to equip attendees with skills in advocacy and policy change.^10^ The workshop utilized case-based learning, using the example of local advocacy by physicians to address precarious employment. We asked students to identify a topic and target for advocacy. Students received two assignments: to participate in a group deputation to a hypothetical policymaker about their topic, and individually to write an opinion editorial. After the workshop, we invited students and facilitators to participate in semi-structured focus groups and provide feedback on the experience of the workshop. We recorded all deputations and focus groups and transcribed them verbatim.

Our analysis focused on understanding how the intervention influenced students, exploring the outputs of the assignments using the lens of structural competency, and to identify opportunities for improvement. Two research assistants coded all transcripts of the deputations and focus groups, and the written assignments, using Microsoft Word (Version 16.22, Microsoft 2019). The five components of Metzl and Hansen’s structural competency paradigm informed our initial coding framework.^14^ Additional codes emerged as two research assistants analyzed the transcripts, and the entire study team identified key themes through consensus.^14^

## Results

We recruited nine medical students to participate in the workshop on policy-level advocacy in October 2017. Eight students completed at least one of the assignments and five students completed both the assignments. Seven of the nine participating students had previous advocacy experience, and all participants identified as women. We will present our findings according to what students chose to do, how the elements of structural competency per Metzl and Hansen were demonstrated (or not) through their assignments, and the students’ feedback on the educational experience.

### What students chose to do

In groups, students performed deputations targeting varied stakeholders. Students required some guidance as to who precisely would be the best target, i.e. would actually have authority over the policy that needed to be changed. One group targeted a municipal Community Development and Recreation Committee seeking improved data collection for deaths among homeless persons. A second group targeted Members of Provincial Parliament seeking expansion of public health insurance to uninsured students. A third group targeted a committee of the federal government regarding national Pharmacare. Opinion-editorial topics included addressing homelessness, improving refugee health, the relationship between opioid misuse and poverty, and addressing domestic violence.

### How structural competency manifested in students’ work

Students produced outputs with appropriately referenced facts about the SDOH paired with narratives that emphasized the importance of the issue. For example, in an opinion editorial about the opioid crisis, the writer identified a higher minimum wage and access to subsidized post-secondary training as structural factors that might have helped prevent one patient’s addiction to opiate medications. The students presented evidence with structural humility, for example, during a deputation a student stated: “*We are not experts in policy or public health, nor have we personally experienced homelessness, but as medical students, we have already observed how gaps in healthcare provision lead to bad outcomes for homeless individuals.”* Students were able to imagine how interventions could address structural causes of ill health and presented potential solutions to barriers that would be faced in both the written and oral outputs.

### Students’ feedback on the educational initiative

Students valued the aspects of the workshop that focused on building skills in oral and written communication. These aspects differentiated the intervention from existing curriculum. One student stated “*We’re often just vaguely taught that advocacy is a CanMEDS role for example, so my expectation was how to actually […] develop that skill, which the session did a good job of doing.”*

Learning the “logistics” of advocacy work, such as how to identify the right stakeholders to engage with and how to approach them, emerged as key to advocacy on SDOH. For example, a participant reflected that *“what could be improved was looking at the logistical sense of advocacy and being aware of the administrative steps.”* Students lacked knowledge on how to depute and how to take an op-ed from an idea through to publication. After the exercise, one student stated: “*How do you even go to a deputation, where do you go, who do you get involved with…”* Another student reflected “*will I ever be able to do this, because I don’t know where to start?”*

## Discussion

In developing this intervention, we focused on applying a structural competency framework as a theory-based guide to advocacy education – one that could provide a foundation of both knowledge and skill – and selected assignments/assessments that could test whether students were able to transfer this knowledge and skill to a relevant structural advocacy situation. We asked students to produce an advocacy output (i.e. a deputation and an opinion-editorial) with some guidance about how to structure these outputs. Student did target structural advocacy as opposed to individual, so this shows some improvement upon prior interventions; students also demonstrated elements of structural competency, lending support to Metzl and Hansen’s framework. However, students lacked some requisite knowledge and were left with a sense of still not knowing what to do.

Our study findings and limitations lead us to important discussion points about the underlying knowledge required as part of advocacy training. Most of our students had previous advocacy experience and already demonstrated an understanding of structural determinants of health; hence our findings may not be generalizable to allmedical students. Our participants all identify as women and are at a similar early point in their training, which might also limit the generalizability of findings. We assumed that medical students had an understanding of the organization of the health system, including political, economic, and social structures that impact the social determinants of health. We also assumed that students would be able to identify the targets of advocacy, including government committees, policymakers, the public or community stakeholders. These assumptions related to underlying knowledge that would be required to engage most effectively with advocacy. Metzl and Hansen’s framework provides a theory about advocacy, but a foundation of knowledge is required for specific acts of advocacy. Future work would need to identify such knowledge and – while perhaps unrealistic to teach all such knowledge within medical school – provide students with paths to acquire such knowledge before engaging in advocacy.

Canadian medical students should graduate capable of communicating with the public and stakeholders (for example, through op-eds and deputations), and capable of identifying appropriate stakeholders at varied levels of government.^1^ However, the structures governing the health system and social determinants of health in Canada are complex and often confusing to health practitioners. In a study by Kuper et. al. on the non-bioscientific knowledge required for medical training, participants suggested that medical learners should be taught about public policy, policy change, and legislative process^15^ Increased educational experiences that include skill-development in navigating and acting on the political and social structures affecting the social determinants of health would need to be integrated with requisite knowledge. For example, Lucey’s proposed addition of social, economic and behavioural sciences as basic sciences required for the development of expertise.^16^

### Conclusion

The next generation of physician advocates require knowledge from many disciplines, fields, and sources, including biomedical, social, personal, and practical. The Metzl and Hansen structural competency framework provides a useful guide for advocacy education focused on advocacy skills. However, given research that demonstrates the importance of basic knowledge in supporting ongoing learning and practice within dynamic systems, we suggest additional attention to the basic knowledge required to engage in specific advocacy efforts.^17^ Advocacy skills training may be required to ensure that medical students can address structural factors perpetuating inequities. Nevertheless, skills training alone is likely insufficient for Canadian medical students to fully embody the role of physician-advocate. Instead, we should teach these practical skills in integration with relevant underlying knowledge about health systems, health policy, and the SDOH.
